# EuroHePP: The European Toolkit to Strengthen National Responses for the Elimination of Viral Hepatitis in Prisons

**DOI:** 10.1111/liv.70874

**Published:** 2026-09-15

**Authors:** Serena Fondelli, Guglielmo Arzilli, Filipa Alves da Costa, Christian Brixko, Maria Butí, Joaquin Cabezas, Nicola Cocco, Olivia Dawson, Charlotte Deogan, Gabriele Fischer, Arkadiusz Komorowski, Fadi Meroueh, Linda Montanari, Sara Mazzilli, Éamonn O'Moore, Roberto Ranieri, Rafaela Rigoni, Alessio Saponaro, Carole Seguin‐Devaux, Ehab Salah, Thomas Seyler, Heino Stöver, Lara Tavoschi, Erika Duffell

**Affiliations:** ^1^ Department of Translational Research and New Technologies in Medicine and Surgery University of Pisa Pisa Italy; ^2^ Research Institute for Medicines (iMED) University of Lisbon Lisbon Portugal; ^3^ WHO Regional Office for Europe Copenhagen Denmark; ^4^ Department of Gastroenterology and Hepatology CHR Citadelle Liège Belgium; ^5^ Liver Unit Hospital Universitario Vall D'hebron and CIBER‐EHD del Instituto Carlos III Barcelona Spain; ^6^ Gastroenterology and Hepatology Department, Clinical and Translational Research in Digestive Diseases, Valdecilla Research Institute (IDIVAL) Marqués de Valdecilla University Hospital Santander Spain; ^7^ Global Prison Infectious Disease Prison Network Sydney Australia; ^8^ ASST Santi Paolo e Carlo Milan Italy; ^9^ The International Network on Health and Hepatitis in Substance Users (INHSU) Geneva Switzerland; ^10^ European Centre for Disease Prevention and Control (ECDC) Stockholm Sweden; ^11^ Center for Public Health Medical University of Vienna Vienna Austria; ^12^ Grüner Kreis Association Vienna Austria; ^13^ PH Chef de Service, Service de Soins en Milieu Pénitentiaire, Pôle Santé Publique et Ecologie de la Santé, CHU de Montpellier Maison D'arrêt de Villeneuve‐lès‐Maguelone France; ^14^ European Union Drugs Agency (EUDA) Lisbon Portugal; ^15^ National Health Protection Office Health Service Executive Dublin Ireland; ^16^ Correlation, European Harm Reduction Network (C‐EHRN) Amsterdam the Netherlands; ^17^ General Directorate of Health and Social Policies Bologna Italy; ^18^ Department of Infection and Immunity Luxembourg Institute of Health Esch‐sur‐Alzette Luxembourg; ^19^ Prisons and HIV, United Nations Office on Drugs and Crime (UNODC) Vienna Austria; ^20^ Institute of Addiction Research Frankfurt University of Applied Sciences Frankfurt Germany

**Keywords:** best practices, micro‐elimination, prison health, test & treat, viral hepatitis

## Abstract

People living in prison are disproportionately affected by hepatitis B and C, yet access to prevention, testing, and treatment services remains inconsistent across Europe. Addressing this gap is critical to achieving viral hepatitis elimination targets by 2030. EuroHePP is a European toolkit developed to support national responses to viral hepatitis in prison settings. It was created through a structured, multi‐stage process involving evidence review, stakeholder consultations, and expert validation. The toolkit provides practical guidance across the full continuum of care, including prevention, testing, treatment, and monitoring, and promotes micro‐elimination approaches tailored to prison populations. It emphasises evidence‐based interventions such as opt‐out testing, decentralised and simplified models of care, harm reduction strategies, and continuity of care after release. By offering adaptable tools and frameworks for policymakers and practitioners, EuroHePP aims to strengthen prison health systems, reduce health inequalities, and accelerate progress towards hepatitis elimination at national and regional levels.

## Key Points

- People in prison bear a disproportionate burden of hepatitis B and C across Europe.
- Prevention, testing, and treatment services remain unevenly implemented in EU/EEA prison systems.
- EuroHePP offers practical guidance across the full continuum of prison‐based hepatitis care.
- Prison‐based micro‐elimination is feasible and accelerates progress towards national elimination targets.

## Lay Summary

People held in prison are far more likely to have hepatitis B and C than the general public, yet they often miss out on testing and treatment. This article introduces EuroHePP, a free European toolkit created by two EU health agencies (ECDC and EUDA) that gives prisons and health authorities practical, ready‐to‐use guidance on preventing, testing for, treating and keeping track of these infections. Making better use of prisons in this way protects the health of people who are incarcerated and helps stop hepatitis spreading into the wider community, bringing Europe closer to eliminating the disease.

**Abbreviations**
BBVs: blood borne viruses
DAA: direct‐acting antiviral
EAG: Expert Advisory Group
ECDC: European Centre for Disease Prevention and Control
EEA: European Economic Area
EU: European Union
EUDA: European Union Drugs Agency
HBV: hepatitis B virus
HCV: hepatitis C virus
NGO: non‐governmental organisations
NSP: needle and syringe programmes
OAT: opioid agonist treatment
PWID: people who inject drugs
PWUD: people who use drugs
UK: United Kingdom
UN: United Nations

## 1 Introduction: Viral Hepatitis in Prison Setting

Chronic viral hepatitis, caused by the hepatitis B (HBV) and C (HCV) viruses, continues to be a major global health concern. The United Nations (UN) Sustainable Development Goals (SDGs) and the World Health Organisation's (WHO) Global Health Sector Strategy have prioritised its elimination as a public health threat by 2030, setting targets of a 65% reduction in hepatitis related deaths and a 90% reduction in the incidence of chronic HBV and HCV infections as compared to the 2015 baseline [1]. The WHO European Action Plan 2020–2025—United Action for Better Health, sets even more ambitious targets, recognising existing systems and prevention efforts [2]. Prioritising the elimination of viral hepatitis is also in line with the European Commission's Beating Cancer Plan, which recommends a focus on strengthening services for the prevention and control of viral hepatitis to reduce liver cancer [3].

Despite this high‐level political commitment, viral hepatitis continues to be a significant burden in the European Union (EU) and European Economic Area (EEA). Recent estimates suggest that there are still approximately 3.2 million people living with chronic HBV infection and 1.8 million people living with chronic HCV infection in the EU/EEA [4, 5]. Importantly, most EU/EEA countries are not on track to meet hepatitis elimination targets, as large proportions of infections remain undiagnosed and untreated [4]. Injecting drug use is a major risk factor for viral hepatitis in Europe, particularly for HCV [6]. Hence, people who use drugs (PWUD), in particular people who inject drugs (PWID), became high priority groups for HCV and HBV elimination in the region. Yet, these groups often face structural barriers to accessing testing, treatment, and harm reduction services [7].

Prisons are a high‐risk environment for HCV transmission, given the high rates of injecting drug use and restricted access to harm reduction interventions, including opioid agonist treatment (OAT) and sterile injecting equipment [8, 9]. PWUD and PWID are disproportionately represented among incarcerated individuals, with estimates suggesting that 58% of PWID have ever been imprisoned, and up to 50% of people in prison in Europe report a history of injecting drug use, the majority of which occurred before imprisonment [9]. Furthermore, initiation of injecting practices may also occur for the first time during incarceration [10]. In a 2020 study of HCV infection in prisons in the EU/EEA and the United Kingdom (UK), seroprevalence of HCV antibodies ranged from 2%–3% to 82%–86%, while viremia ranged from 5%–7% to 82% [11].

People in prison bear a disproportionate burden of HBV infection, largely reflecting the higher prevalence among PWID compared with the general population [12]. Across the EU/EEA, foreign nationals constitute an average of 25% of the prison population [13], many originating from countries with high HBV endemicity and low vaccination coverage, which further contributes to the elevated prevalence observed in prison.

A recent systematic review of studies conducted in EU/EEA countries reported that the prevalence of HBV infection among people in prison ranged from 0.6% in France to 8.3% in Greece [12]. Overcrowded living conditions, suboptimal infection control practices and increased frequency of high‐risk behaviours, such as sharing injecting equipment, unsafe tattooing and piercing practices and unprotected sex, are also risk factors that contribute to an increased risk of viral hepatitis transmission [14, 15]. Moreover, many people in prison come from socio‐economically disadvantaged communities, often experiencing constrained access to healthcare services before incarceration [16].

In 2023, the European Centre for Disease Prevention and Control (ECDC) collected data related to the prevention and control of HBV and HCV across EU/EEA countries, including health services in prisons [17]. Out of the 30 responding countries, only 19 reported having a national HBV testing policy or programme for people in prison, and 21 reported having such a policy or programme for HCV. Complemented by other epidemiological data, these findings suggest that hepatitis B and C prevention and control services may not be widely implemented in many European countries [18, 19, 20, 21].

Beyond the public health significance, efforts towards control of hepatitis B and C in prison settings represent a significant step forward in upholding the equity and human rights of marginalised populations. Previous ECDC and European Union Drugs Agency (EUDA) public health guidance has also highlighted the importance of comprehensive interventions for the prevention and control of blood borne viruses (BBVs) in prison settings, including testing, active case finding and linkage to care [22]. In the context of prison health, the principle of equivalence of care is most prominently articulated in the United Nations Standard Minimum Rules for the Treatment of Prisoners (the Nelson Mandela Rules, 2015), which claims that ‘prisoners should enjoy the same standards of health care as are available in the community and should have access to necessary health care services free of charge without discrimination on the grounds of their legal status’ (Rule 24) [23].

Without tailored and effective services for active case finding and linkage to care for people in prison, broader community efforts to eliminate blood‐borne viruses (BBVs), like hepatitis, may be undermined. As prisons are an integral part of our communities and their populations are highly dynamic, the absence of adequate interventions allows them to act as amplifiers of transmission, sustaining infection both in prison and community settings.

Supporting the enhancement of viral hepatitis elimination efforts within the prison system is paramount. To assist European countries in scaling up targeted interventions in prison populations and support the implementation of existing guidance on BBV prevention and control, with a specific focus on viral hepatitis elimination, the ECDC and the EUDA have developed the European Toolkit for the Elimination of Viral Hepatitis in Prisons (EuroHePP), designed to guide and strengthen national responses within the prison system (accessible at: https://eurohepp.ecdc.europa.eu) [24]. The objective of EuroHePP is to support the scale‐up of services for the prevention and control of HBV and HCV in prison settings, tailored towards people working in prison, prison health authorities and policymakers, building upon existing evidence and practices. While the primary focus is the European region, its framework may be adapted and relevant for use in all other regions. The aim of this paper is to promote EuroHePP and its recommended practices as a resource to inform and complement guidelines [25].

## 2 Methodology: The Development of the European Toolkit

### 2.1 Collaborative Framework

A multi‐institutional collaborative approach was used to develop the online toolkit under the joint leadership of the ECDC and EUDA, and with the support of a consortium comprising the University of Pisa, the International Network on Health and Hepatitis in Substance Users (INHSU) ‐ Prisons Network, and the Kirby Institute of the University of New South Wales. The consortium's structure ensured comprehensive expertise in epidemiology, viral hepatitis management, and prison health.

An expert advisory group (EAG) was established to provide scientific guidance and input, comprising professionals from national institutes of public health, healthcare services, academia, UN agencies, other international agencies, learned societies, and civil society organisations.

### 2.2 Toolkit Development

The methodology followed a systematic four‐stage process: (1) Scoping and Objectives: Initial survey and focus groups were conducted among ECDC/EUDA expert networks to identify barriers and facilitators for the implementation of evidence‐based interventions to control hepatitis B and C in prison settings. The results of this work provided information on key areas to be addressed in the toolkit; (2) Evidence identification: identification of evidence‐based information sources, clinical guidelines, policy frameworks, and best‐practice resources for inclusion in the toolkit, including data on barriers to care, supporting evidence from the literature, findings from European projects (such as practical tools to guide implementation), and models of care implemented in European countries; (3) Toolkit Drafting: iterative development of draft user‐friendly and action‐oriented toolkit components and compilation of evidence‐based tools; and (4) Feedback and validation: structured feedback collection from the EAG through remote review and virtual consultations.

**FIGURE 1:**
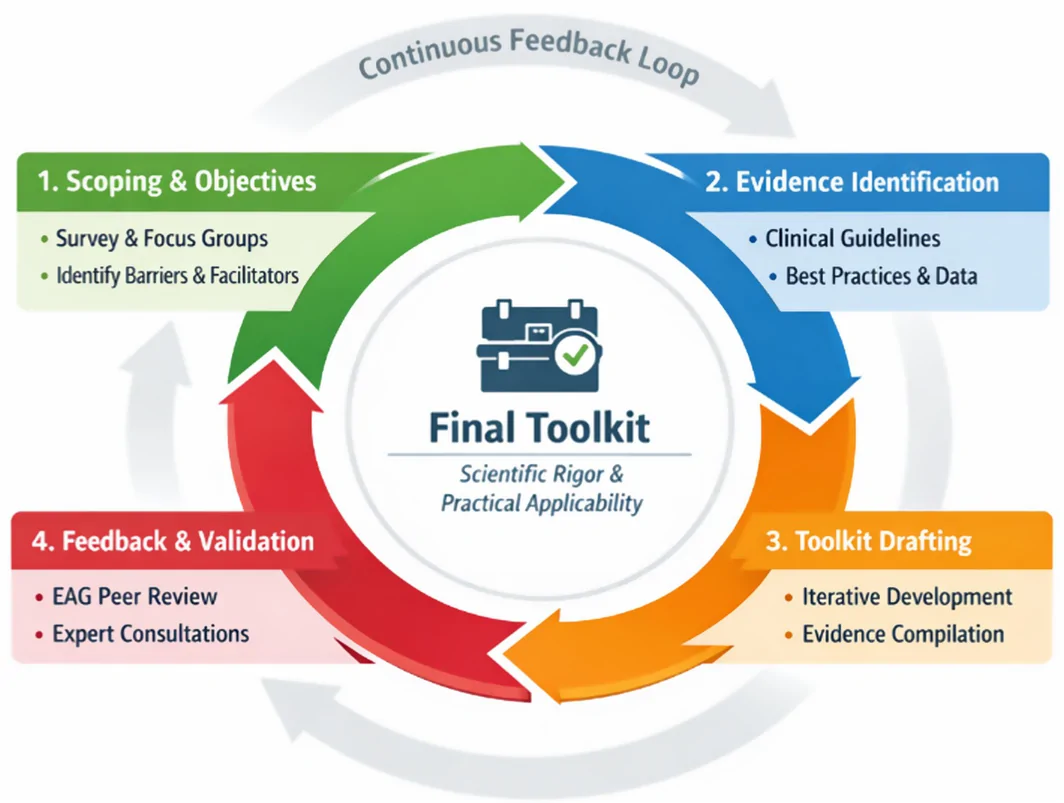
Four‐stage process explaining the toolkit development.

The methodology incorporated continuous feedback loops between the project team and the EAG to ensure scientific rigour (e.g., by identifying the most recent and relevant evidence in the field) and practical applicability. All toolkit components underwent peer revie w by subject matter experts within the EAG. Figure 1 reports this four‐stage process.

## 3 Results: A Toolkit to Support the Elimination of Viral Hepatitis B and C in Prisons

### 3.1 Aim and Structure of the Toolkit

**FIGURE 2:**
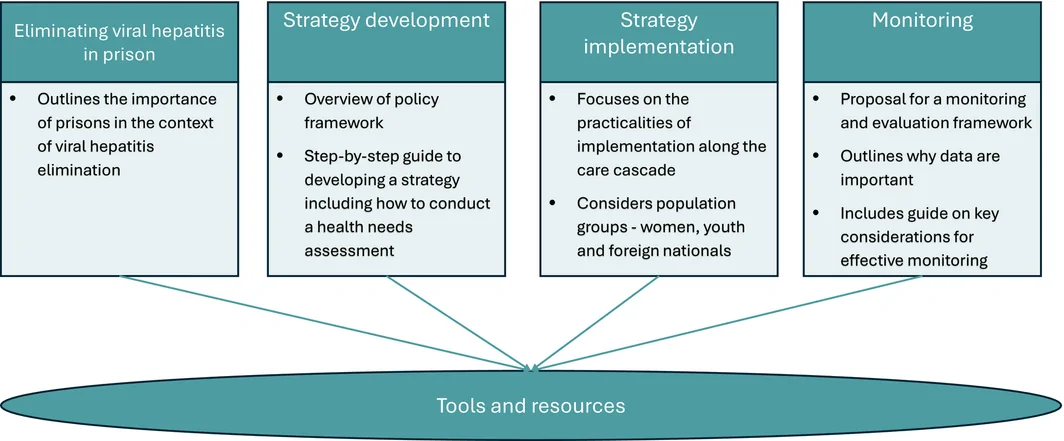
Structure of the European toolkit to support the elimination of viral hepatitis B and C in prisons.

The structure identified as most suitable was one incorporating four interconnected components and integrating multiple sources of information and useful materials gathered through the ‘Evidence Identification’. Together, these components offer a comprehensive and practical resource for health care providers working in prison settings, along with people working in prison, prison health authorities and policymakers seeking to enhance national responses to hepatitis B and C in prison settings (Figure 2).

– *Component 1: Eliminating viral hepatitis in prisons*
  This component describes the epidemiological and political context and highlights the importance of prisons within the general viral hepatitis elimination agenda
– *Component 2: Strategy development*
  This component provides an overview of the relevant policy frameworks and presents a structured, step‐by‐step guide for developing elimination strategies
– *Component 3: Strategy implementation*
  This component addresses the practical aspects of delivering hepatitis services within the constraints of the prison system
– *Component 4: Monitoring*
  This component provides a framework for monitoring and evaluation of prison‐based viral hepatitis elimination efforts.

### 3.2 Component 1: Eliminating Viral Hepatitis in Prison

Given the disproportionately high burden of hepatitis B and C among incarcerated populations, EuroHePP highlights prisons as critical settings for targeted prevention, testing, and treatment efforts. It provides a robust rationale for political commitment and concerted actions [24]. Prison settings represent an important platform for healthcare service delivery for individuals who might not otherwise access community services, owing to limited awareness or barriers to healthcare access. By reaching populations that are typically underserved, prisons can contribute to reducing health inequalities [26].

In this context, the concept of micro‐elimination offers a pragmatic framework for achieving national elimination targets through focused interventions in defined population groups [27]. The micro‐elimination approach is rooted in the identification and implementation of person‐centred care models based on the specific needs and risk profiles of distinct population groups [28, 29].

There is strong evidence supporting viral hepatitis elimination programmes in prisons [11, 30, 31, 32, 33] that typically include universal testing programmes, early access to effective antiviral treatment, harm reduction strategies and linkage to post‐release care [34, 35]. Studies and pilot programmes in some European countries have demonstrated the feasibility and effectiveness of such initiatives [30, 33, 36]. Extensive evidence developed in Australia and elsewhere demonstrate the feasibility of HCV elimination in prison settings, using opt‐out universal testing programmes alongside adapted and decentralised care models [37, 38, 39]. The scaling up of these programmes has shown significant reductions in the incidence of viral hepatitis in prison populations, providing compelling evidence for the use of universal test‐and‐treat approaches as a primary prevention strategy in this setting [40].

### 3.3 Component 2: Strategy Development

This component of the toolkit guides the development of elimination strategies through a structured, stepwise approach, from initial planning to finalisation. It provides a set of tools and templates tailored to the prison setting to conduct needs assessment, identifying service gaps and adapt interventions to the specific local contexts.

The capacity to implement and scale up viral hepatitis services in prisons is strongly influenced by the regional, national, and local political and economic context. This context affects multiple factors, including the availability and accessibility of harm reduction services and other care strategies both within prisons and in the community, restrictions on medical specialities authorised to prescribe treatment, eligibility criteria for hepatitis therapy, funding for vaccinations or treatment programmes, and the local availability of antiviral drugs for hepatitis B and C. Additionally, policies that restrict access to care for specific groups, such as individuals who continue to use drugs, can institutionalise and perpetuate stigma against marginalised populations. This stigmatisation may create further barriers, deterring individuals from seeking diagnosis and treatment due to fear of discrimination from correctional staff or other people living in prison [41].

The development of a viral hepatitis elimination strategy involves a structured process that begins with the identification of internal stakeholders (e.g., prison administration, prison officers, prison health services, people living in prison) and external stakeholders (public health officials, civil society organisations, community health organisations, research institutions, non‐governmental organisations). Then, a multidisciplinary prison hepatitis elimination steering group is established. This is followed by a health needs assessment of the prison setting, including mapping existing services and characterisation of the prison population, which informs the identification of required interventions [42]. The assessment enables the prioritisation of health needs and the design of services across the care cascade (from prevention, testing, treatment and aftercare) tailored to the context and grounded in a person‐centred approach.

Where the legislative or policy environment does not support the effective provision of hepatitis and/or harm reduction services, advocacy is essential [43]. Advocacy efforts are necessary to align policy with strategy and ultimately service provision. The uneven coverage of existing services highlights a substantial opportunity to develop and expand local, regional and national policy frameworks across Europe to accelerate progress towards hepatitis elimination. Several tools are available to support these efforts; for instance, INHSU has developed advocacy tools for the promotion of acceptable and more inclusive prison health policies [42, 44]. Person‐centred care, which emphasises the needs, preferences, and values of individuals and actively involves them in decision‐making, is recognised by WHO as essential for primary healthcare and Universal Health Coverage. In prisons, this approach helps address unique challenges such as stigma, discrimination, and barriers to healthcare access [45].

### 3.4 Component 3: Strategy Implementation

This component of EuroHePP addresses the practical aspects of delivering hepatitis services along the entire care cascade, from prevention, screening and diagnosis to treatment and follow‐up, within the constraints of the prison system.

#### 3.4.1 Prevention Interventions

Unsafe injecting practices among PWID remain a major driver of HCV and other BBVs transmission [46]. The implementation of comprehensive and combined harm reduction interventions could have a considerable impact in prison settings.

Needle and Syringe Programmes (NSPs) provide access to sterile equipment (e.g., needles, syringes, and drug preparation equipment) with the primary aim of reducing the transmission of infections among PWID. In 2023, NSP programmes were formally available in prisons in only four European countries, although not consistently across all facilities [8]. Whilst evidence on NSPs in European prison settings is limited, it is consistent in supporting its effectiveness and feasibility [47, 48]. Recently published global guidelines have stressed that NSPs in prisons are essential to viral hepatitis elimination. These recommendations highlight that, in the absence of robust prison‐specific trials, findings from large observational cohort studies of people who inject drugs in community settings provide an appropriate and ethically justified basis for policy and practice in custodial environments [49].

Another key harm reduction intervention to reduce hepatitis C transmission is ensuring the availability of opioid agonist therapy (OAT) in prison. Individuals already on OAT before entering prison should be able to continue with treatment. OAT may also be initiated in prison, and should then be continued upon release, through the establishment of links with outside community services. There is evidence to suggest that providing OAT during incarceration reduces injecting‐related risks and increases engagement with community treatment after release. Individuals who continue OAT post‐release are less likely to experience drug‐related deaths and show reduced all‐cause mortality [22, 50, 51, 52].

Whenever possible, interventions should be combined to achieve synergistic effects. Evidence from existing models of care described in EuroHePP indicates that harm reduction activities in prison are successful when integrated with educational initiatives targeting both people in prison and prison staff, to ensure awareness of preventive measures. In addition, harm reduction is frequently combined with social support, which is particularly critical to ensure continuity of care after release. Such support may include the involvement of psychologists and social workers, housing programmes, and other reintegration services, all of which contribute to reducing post‐release vulnerability and improving health outcomes.

Other evidence‐based interventions to prevent BBVs should be considered for implementation in prisons: HBV vaccination, access to antiviral treatment both to slow disease progression and to prevent further transmission, safe tattooing and body piercing, condom distribution, pre‐ and post‐exposure prophylaxis, and prevention of mother‐to‐child transmission [22, 53].

The HBV vaccine is highly effective in preventing HBV infection and thereby reducing the reoccurrence of the complications of infection including chronic liver disease, cirrhosis, and HBV‐related hepatocellular carcinoma [54]. Although most European countries have implemented childhood HBV vaccination programmes with high coverage [55], some groups may remain insufficiently reached, particularly underserved populations, including people in prison and some migrant communities. While HBV vaccination programmes are implemented in many European prisons and recognised as effective [22], important gaps persist in services availability, standardisation, and data on coverage and monitoring across the region [56, 57].

Serological testing (HBsAg, anti‐HBs, anti‐HBc) before vaccination can identify individuals with existing immunity or active HBV infection who do not require vaccination. However, pre‐vaccination testing should not delay vaccination of those potentially susceptible, particularly where exposure risk is high. If testing is performed, the first vaccine dose may be administered at the time of blood collection. If results confirm immunity or active infection, no further doses are needed. Where testing is not feasible, hepatitis B vaccination should be provided without delay.

Given the transient nature of people in prison, vaccination should be delivered as early as possible during contact with services. The standard adult schedule (0, 1, and 6 months) is often difficult to complete in custody. Accelerated schedules (e.g., Days 0, 7, and 21, or Months 0, 1, and 2) may therefore be used, with or without a fourth dose at Month 12 [58]. A recently licensed HBV vaccine requires only two doses over 1 month and may be considered where available.

#### 3.4.2 Testing Interventions

Testing in prisons is an effective strategy to expand testing uptake among key populations. Active case finding should be offered voluntarily using either opt‐in testing (where individuals choose whether to be tested) or opt‐out testing (where testing is performed unless actively refused) approaches [59]. Although opt‐in testing and risk‐based testing are commonly implemented in European prisons, modelling studies suggest that opt‐out testing is both more effective and cost‐effective [60, 61, 62].

While venepuncture remains standard practice in many EU countries, venous access can be difficult for individuals with a history of injecting drug use, and long laboratory turnaround times can be problematic. Alternative approaches, such as rapid antibody tests using saliva samples, point‐of‐care testing requiring finger‐prick blood and dried blood spot testing, are increasingly available and have proven effective in prison settings due to their ease of use and rapid results [35]. Point‐of‐care testing enables detection of active infection within minutes to hours, facilitating prompt treatment initiation, reducing the need for multiple follow‐up visits, and improving adherence.

An integrated testing approach is particularly relevant in prison settings, where individuals at risk of one blood‐borne infection are also more vulnerable to others. A coordinated, multi‐infection strategy is therefore recognised as more effective and efficient.

#### 3.4.3 Treatment Interventions

For both chronic HBV and HCV infections, highly effective antiviral treatment exists that can reduce the progression to cirrhosis, the risk of hepatocellular carcinoma and liver‐related deaths.

The introduction of highly effective, short‐duration, direct‐acting antiviral (DAA) therapies has revolutionised HCV treatment, with cure rates above 95% [63]. ECDC and EUDA guidance recommends pan‐genotypic regimens that do not require pre‐treatment genotype testing for populations such as people in prison, where a streamlined care pathway is preferable. When treatment cannot be completed during incarceration, the preferred approach is to ensure continuity of care through effective referral to community service. There may be legal barriers in the transition prison/community regarding access to viral hepatitis services for non‐resident individuals. Providing patients with instructions around the treatment by a healthcare professional and giving the remainder of their medication upon release may be considered as a secondary option when referral is not feasible.

Individuals with chronic hepatitis B should be assessed for treatment, and those who are eligible can receive therapy to slow the progression of cirrhosis, reducing the risk of liver cancer and improving long‐term survival [64]. Most people who start hepatitis B treatment require lifelong therapy, which should not be discontinued without medical assessment. For this reason, ensuring continuity of care is essential for individuals receiving treatment in prison, so that once they are released, the cascade of care is not disrupted and loss to follow‐up is minimised.

#### 3.4.4 Best Practice for Intervention Implementation

The EuroHePP toolkit offers a range of dedicated resources, including the EUDA Best Practice portals for prison‐based interventions [65], examples of effective models of care implemented in various European countries, and recommendations derived from evidence‐based guidance documents. These resources consistently highlight key elements that have been shown to contribute to the successful implementation of interventions.

Lack of knowledge among healthcare and prison staff regarding harm reduction and hepatitis B and C interventions contributes to stigma within prisons and can hinder testing and treatment uptake. Task shifting, which involves delegating specific healthcare tasks to less specialised providers, is crucial in resource‐limited settings such as prisons, where shortages of highly trained personnel can limit the delivery of high‐quality care. Training staff to perform these tasks not only enhances treatment capacity but also helps reduce stigma by increasing knowledge and engagement. Support can come from non‐specialised healthcare staff, as well as from primary care physicians and nurses.

The implementation of peer‐support programmes, facilitated by individuals with lived experience of hepatitis or imprisonment, can play a pivotal role in disseminating accurate health information, raising awareness, and promoting prevention. Drawing on shared experience, peers can effectively engage people in prison, address concerns, and dispel misconceptions, thereby improving treatment uptake and adherence. These programmes also address psychosocial needs, helping to reduce hepatitis‐related stigma and providing a platform for shared experiences. Furthermore, peer‐led promotion of harm reduction practices, such as safer injecting, contributes to a safer prison environment.

The use of interdisciplinary teams delivering integrated services, including medical staff, social workers, psychologists, and, where appropriate, legal advisors, enables comprehensive, patient‐centred care. This collaborative approach supports coordinated clinical management, improves continuity of hepatitis care, and helps address the complex and often overlapping health and social needs of people in prison.

Continuity of care beyond the prison walls remains a critical challenge. Establishing solid communication channels, continuity of care protocols, and shared care plans between prison facilities and community healthcare services is essential to bridge gaps in clinical information and prevent treatment disruptions [66]. Active engagement with community organisations, including NGOs, civil society organisations, and support groups, can further support seamless transitions, strengthen continuity of care, and promote a more comprehensive, person‐centred approach.

The implementation of electronic medical record systems with secure, real‐time data transfer capabilities between prison healthcare units, external laboratories, and community healthcare services, while maintaining patient confidentiality, can facilitate seamless exchange of clinical information, support continuity of care during incarceration and after release, and improve coordination between prison and community‐based healthcare providers.

When implementing viral hepatitis testing and treatment, it is important to have an intersectional perspective in order to take into consideration specific discriminatory factors. People in prison have diverse and overlapping identities encompassing age, gender, sexual orientation, migrant background and social context. That shapes their experiences of hepatitis‐related stigma. This stigma can compound challenges for certain subgroups. Understanding these nuances is crucial for tailoring interventions to the unique barriers faced by different identities. Examples of an intersectional approach may include, but are not limited to, integrating specific care for pregnant women, such as preventing vertical hepatitis transmission, addressing cultural and linguistic diversity, and ensuring healthcare access for people with a migrant background through cultural mediators.

### 3.5 Component 4: Monitoring and Evaluation Systems to Assess Progress

The fourth component emphasises the importance of strategic monitoring to assess progress, ensure accountability, identify service gaps, and support the implementation of targeted interventions aimed at achieving hepatitis elimination in prisons. The considerable heterogeneity of prison health policies across European countries underscores the need for systematic data collection to enable effective monitoring and evaluation of the impact of different approaches [67].

Prisons should have a well‐functioning health information system to generate timely data to monitor the health status of the prison population through well‐defined epidemiological indicators and evaluate the performance of the prison health system [68, 69]. The information system should be able to support the sharing of health information across prisons and their integration with health systems in the community (including those used in ambulatory care and in hospitals, involving both public and private sectors). This system should be designed to support individual clinical care as well as to monitor hepatitis B and C (among other conditions) along the continuum of care and to be integrated with other programmes, including vaccination programmes, OAT, NSPs, and other harm reduction and treatment interventions.

The toolkit includes information on case definition, a template for case reporting, and key considerations for designing effective monitoring systems. Moreover, it offers guidance on selecting relevant indicators to track implementation and health outcomes. A set of core indicators was identified in line with the regional monitoring framework (Table 1) [70]. The indicators reflecting prevention include hepatitis B vaccine coverage, sterile syringes and needle sets distributed to persons who inject drugs, and OAT coverage. Other core indicators reflect the cascade of care and include screening offer, screening uptake and coverage for both infections among the prison population, the number (and proportion) of those screened who are diagnosed with chronic infection, the coverage of care/treatment, the effectiveness of treatment and continuity of care following release or transfer.

**TABLE 1:** Description of the core indicators and associated data for monitoring progress towards hepatitis elimination in prisons.

Area	Indicator	Numerator	Denominator	Method
Burden	Proportion of people in prison with lifetime prevalence of drug injecting Proportion of people in prison with lifetime prevalence of drug injecting inside prison Proportion of people in prison with chronic HBV infection (HBsAg positive)	Number of people living in prison who have experience of drug injection Number of people living in prison who have experience of drug injection inside prison Number of persons in prison who tested positive for chronic HBV infection (HBsAg) during the reporting period (or at a given point in time)	Number of people living in prison Number of people living in prison Number of persons in prison who were tested for chronic HBV infection (HBsAg) during the reporting period (or at a given point in time)	Cross‐sectional survey using the European Questionnaire on Drug Use among People living in prison Cross‐sectional survey using the European Questionnaire on Drug Use among People living in prison Routinely collected administrative data or ad hoc survey
	Proportion of people in prison with chronic HCV infection (HCV RNA or HCV‐Ag positive)	Number of persons in prison who tested positive for chronic HCV infection (HCV RNA or HCV‐Ag positive) during the reporting period (or at a given point in time)	Number of persons in prison who were tested for HCV infection (anti‐HCV, HCV RNA or HCV‐Ag) during the reporting period (or at a given point in time)	Routinely collected administrative data or ad hoc survey
	Proportion of people in prison with past exposure to HCV (anti‐HCV positive)	Number of persons in prison who tested positive for past exposure to HCV (anti‐HCV positive) during the reporting period (or at a given point in time)	Number of persons in prison who were tested for past exposure to HCV (anti‐HCV positive) during the reporting period (or at a given point in time)	Routinely collected administrative data or ad hoc survey
Transmission	New HBV infections (proxy for incidence)	Number of new HBV infections occurring in prison during the reporting period (negative test followed by positive test; i.e., documented seroconversion)	To get incidence rate, the number of new infections should be divided by the prison population during the reported period	Routinely collected administrative data
	New HCV infections (proxy for incidence)	Number of new HCV infections occurring in prison during the reporting period (negative test followed by positive test; i.e., documented seroconversion)	To get incidence rate, the number of new infections should be divided by the prison population during the reported period	Routinely collected administrative data
Prevention	HBV vaccine coverage	Number of persons in prison with complete hepatitis B vaccination (or evidence of immunity)^a^ at a given point in time	Number of persons in prison at a given point in time	Routinely collected administrative data or ad hoc survey
	Harm reduction for people who inject drugs: coverage of needles–syringes^b^ *Alternative indicator in absence of data: availability of NSP in prison*	Number of sterile needles–syringes distributed during the reporting period	Number of persons who inject drugs in prison during the reporting period *Alternative denominator: number of persons in prison*	Routinely collected administrative data or ad hoc survey
	Harm reduction for people who use drugs: coverage of opioid agonist therapy^c^ *Alternative indicator in absence of data: availability of OAT in prison*	Number of persons in prison receiving opioid agonist therapy during the reporting period (or at a given point in time)	Number of persons who use opioids during the reporting period (or at a given point in time) *Alternative denominators: number of persons who inject opioids or number of persons who use NSP programmes or number of persons in prison*	Routinely collected administrative data or ad hoc survey
Continuum of care	Hepatitis B screening coverage	Number of new arrivals to prison who were tested for hepatitis B during the reporting period (e.g., 1 year) using HBsAg testing	Number of new arrivals to the prison over the course of the reporting period^c^	Routinely collected administrative data or ad hoc survey
	Hepatitis C screening coverage	Number of new arrivals in prisons who were tested for past exposure to hepatitis C during the reporting period (e.g., 1 year) using anti‐HCV testing	Number of new arrivals to the prison over the course of the reporting period^c^	Routinely collected administrative data or ad hoc survey
	Hepatitis C diagnostic test coverage	Number of persons in prison who were tested for chronic hepatitis C infection during the reporting period using HCV RNA testing or HCV‐antigen	Number of persons in prison who tested positive for anti‐HCV during the reporting period	Routinely collected administrative data or ad hoc survey
	Test positivity hepatitis B	Number of persons in prison who tested positive for hepatitis B during the reporting period using HBsAg testing	Number of persons who were tested for hepatitis B during the reporting period using HBsAg testing	Routinely collected administrative data or ad hoc survey
	Test positivity hepatitis C	Number of persons in prison who tested positive for HCV RNA (active infection) during the reporting period	Number of persons who were tested for HCV RNA during the reporting period	Routinely collected administrative data or ad hoc survey
	HBV treatment coverage	Number of persons in prison initiated on treatment for chronic HBV infection during the reporting period	Number of persons in prison who tested positive for hepatitis B during the reporting period using HBsAg testing	Routinely collected administrative data or ad hoc survey
	Proportion of people diagnosed with HCV initiated on treatment	Number of persons in prison with chronic HCV infection who *initiated* treatment during a specified time frame	Number people living in prison diagnosed with chronic HCV infection (HCV RNA) during the reporting period	Routinely collected administrative data or ad hoc survey
	Proportion of people in prison with chronic HBV infection on treatment with suppressed HBV viral load	Number of persons in prison with chronic HBV infection on treatment who have achieved suppressed viral load, based on HBV viral load measurement during the reporting period	Number of patients with chronic HBV infection on treatment and assessed for HBV viral load during the reporting period whilst in prison	Routinely collected administrative data or ad hoc survey
	Proportion of people in prison who completed treatment for hepatitis C who were cured	Number of people who completed HCV treatment whilst in prison, during the reporting period, and achieved a sustained virologic response based on viral load measurement at 12 or 24 weeks after the end of treatment	Number of people who completed antiviral treatment for HCV whilst in prison, during the reporting period, and were assessed for sustained viral response at 12 or 24 weeks after the end of treatment	Routinely collected administrative data or ad hoc survey

	Indicator	Assessment question	Method
	Linkage to care for cases of hepatitis B on treatment	Is there a strategy in place to guarantee treatment continuity after release from prison? (yes/no) Are patients treated for HBV in the prison provided with a letter or other communication to their normal care provider in the community concerning their diagnostic or therapeutic information to support continuity of care?	Routinely collected administrative data or ad hoc survey
	Linkage to care for cases of hepatitis C on treatment	Is there a strategy in place to guarantee treatment continuity after release from prison (if needed)? (yes/no) Are patients treated for HCV in the prison provided with a letter or other communication to their normal care provider in the community concerning their diagnostic or therapeutic information to support continuity of care?	Routinely collected administrative data or ad hoc survey

^a^ Either given a full course of HBV vaccination in prison or with documentation that previously fully vaccinated against HBV or not susceptible to infection based on serological results.

^b^ Alternative is policy information on whether needles and syringes or opioid agonist treatment is available.

^c^ Where possible, the denominator should exclude all those with documentation of a recent hepatitis test (i.e., within the last year).

Having the ability to support the implementation of a functional monitoring system that is inclusive of prisons is essential for all countries to understand where additional investments are needed to achieve elimination targets. Excluding people living in prisons would result in an incomplete and inaccurate assessment of national and international progress. The toolkit proposes that monitoring can occur at multiple levels: local (prison), regional, and ideally national, allowing for scale‐up as data systems and resources permit. While the proposed monitoring framework is ambitious, it represents a gold standard designed to guide countries towards comprehensive and effective hepatitis elimination strategies. Therefore, the capacity to upscale information systems to extract the proposed indicators is crucial to overcome data gaps and enable an accurate assessment of progress towards viral hepatitis elimination [71].

## 4 Limitations

This study has some limitations. First, although the toolkit was developed through a structured process informed by the available evidence and expert input, it has not yet undergone formal implementation or impact evaluation in real‐world settings. Consequently, its effectiveness, feasibility, and influence on practice remain to be established through prospective validation studies. Second, as the primary objective of this work was to describe the development of the toolkit, detailed methodological information regarding the evidence synthesis process and expert consensus procedures is not reported in full.

## 5 Conclusions

Prisons bear a disproportionate burden of viral hepatitis, making them a critical setting for achieving elimination targets. Expanding prevention, testing, treatment, and harm reduction services in prisons is essential to reduce transmission, improve individual health outcomes, and generate broader public health benefits and cost savings. Comprehensive, integrated care for hepatitis B and C in prison settings is key to preventing reinfection, managing complications, and advancing elimination goals. Far from being solely a custodial health measure, prison‐based interventions are an integral component of a broader public health strategy: by ensuring continuity of care as individuals transition between the prison and the community, they promote health equity, reduce transmission, and support national elimination targets.

A clear understanding of objectives and timelines for the elimination of viral hepatitis enables governments and prison authorities to define and implement practical, context‐specific actions. Effective implementation requires adequate resources, including a trained workforce and robust information systems. The use of a defined set of indicators ensures interventions remain adaptable to changing contexts and emerging needs, guiding progress towards viral hepatitis elimination.

The toolkit for the elimination of viral hepatitis in prisons, developed by the ECDC and the EUDA, provides a practical resource for professionals working in European prison settings, presenting best practices alongside real‐world examples of successful interventions, highlighting their feasibility and effectiveness in designing and implementing micro‐elimination strategies within prisons.

## Author Contributions

S.F., G.A., E.D. and L.T. conceived and designed the study. S.F. and G.A. drafted the first version of the manuscript. S.M., M.B., N.C., F.M., R.R., R.R., J.C., F.A.C., C.D., C.B., O.D., A.K., G.F., É.O'.M., E.S., A.S., C.S.‐D. and H.S. critically revised the manuscript for important intellectual content. E.D., L.T., L.M., T.S. and F.A.C. supervised the study and overall project activities. L.T. and E.D. acquired funding. All authors reviewed, read, and approved the final version of the manuscript.

## Funding

This work was funded by the European Centre for Disease Prevention and Control (ECDC) under Service Contract No. ECD.14789/2020 for the project ‘Support development of a joint ECDC–EMCDDA toolkit for the elimination of hepatitis in the prison setting’.

## Conflicts of Interest

Ehab Salah alone is responsible for the views expressed in this publication and these do not necessarily represent the decisions or the stated policy of UNODC. Filipa Alves da Costa worked as a consultant for the WHO Regional Office for Europe during the development of this toolkit. The author alone is responsible for the views expressed in this publication, and these do not necessarily represent the decisions or the stated policy of the World Health Organisation.

## Data Availability

Data sharing not applicable to this article as no datasets were generated or analysed during the current study.
